# Association between lower serum bicarbonate and renal hyperfiltration in the general population with preserved renal function: a cross-sectional study

**DOI:** 10.1186/s12882-015-0218-y

**Published:** 2016-01-06

**Authors:** Minseon Park, Rina So, Kwon Wook Joo, Hyung-Jin Yoon

**Affiliations:** Department of Family Medicine, Seoul National University Hospital, Seoul, Republic of Korea; Department of Biomedical Engineering, Seoul National University College of Medicine, 101 Daehak-ro, Jongno-gu, Seoul, 110-744 Republic of Korea; Department of Internal Medicine, Seoul National University College of Medicine, Seoul, Republic of Korea

**Keywords:** Chronic kidney disease, Glomerular filtration rate, Metabolic acidosis, Renal hyperfiltration, Serum bicarbonate

## Abstract

**Background:**

Lower serum bicarbonate, mainly due to the modern Western-style diet, and renal hyperfiltration (RHF) are both independently associated with higher mortality in the general population with preserved renal function. The objective of this study was to evaluate the association between serum bicarbonate and RHF.

**Methods:**

The health data of 41,886 adults with an estimated glomerular filtration rate (eGFR) ≥60 mL/min per 1.73 m^2^ were analyzed. The eGFR was calculated with the Chronic Kidney Disease Epidemiology Collaboration creatinine equation and RHF was defined as eGFR with adjusted residuals > sex-specific 95^th^ percentile.

**Results:**

The adjusted mean of eGFR was lower in the highest quintile of serum bicarbonate than in other quintiles, after adjusting for confounders. A lower percentile rank of serum bicarbonate was associated with higher odds of RHF. The odds ratio (OR) for RHF in the lowest quintile of serum bicarbonate was 1.39 (95 % confidence interval, 95 % CI, 1.11–1.75) compared to the highest, after adjusting for confounders. With subgroup analysis, the association was prominent in participants with a body mass index >25 kg/m^2^ (OR 1.98, 95 % CI 1.32–2.95 in the lowest quintile compared to the highest), compared to those with a body mass index ≤25 kg/m^2^ (OR 1.18, 95 % CI 0.89–1.56 in the lowest quintile compared to the highest).

**Conclusions:**

This study observed an association between lower serum bicarbonate and higher odds of RHF and the possible differential effect of obesity in this association. It is necessary to confirm the association between lower serum bicarbonate and RHF and its causality.

## Background

Metabolic acidosis is one of the earliest complications of chronic kidney disease (CKD). Low serum bicarbonate has been associated with a poor renal outcome and increased mortality, while the beneficial effects of raising serum bicarbonate with alkali supplementation or dietary interventions have been reported in patients with CKD [1]. The correlation between low serum bicarbonate and an increased risk of incident CKD has been reported in community-dwelling cohorts [2, 3]. Although several mechanisms explaining the association between low serum bicarbonate and poor renal outcome in patients with CKD [1] have been suggested, the underlying mechanism explaining the association between low serum bicarbonate and incident CKD in the general population is not yet clear.

Renal hyperfiltration (RHF), which may be a potentially reversible stage of CKD, has been associated with many clinical conditions such as diabetes, hypertension and obesity, and with various lifestyle factors such as smoking, low cardiopulmonary fitness, and a lack of physical activity [4–11]. Recently, we have reported an association between RHF and increased all-cause and cardiovascular mortality in a relatively healthy adult population [12].

An acidogenic diet, deficient in fruit and vegetables, coupled with excessive consumption of animal products and sodium chloride, has been associated with cardiovascular risk by causing insulin resistance, an elevation in blood pressure, and metabolic syndrome. RHF has been proposed as one of the renal adaptive responses to an acidogenic diet, which is believed to be the main cause of low serum bicarbonate in subjects with preserved renal function [13]. The correlation between RHF and low serum bicarbonate has not been tested, however. Elucidating the association between RHF and low serum bicarbonate may be important for studying the pathophysiology of linkage between dietary factors, CKD and cardiometabolic risk and for developing dietary guidelines for the prevention of CKD and long-term, all-cause and cardiovascular mortality associated with RHF in the general population.

To evaluate the relationship between the serum bicarbonate level and RHF, we examined the health screening data of a relatively healthy population of 41,886 adults with an estimated glomerular filtration rate (eGFR) of 60 mL/min per 1.73 m^2^ or higher.

## Methods

### Participants and data collection

Among 68,838 health screenings performed at the Health Promotion Centre of Seoul National University Hospital between Jan 2001 and Dec 2012, 14,465 repeated screenings and 10,577 screenings with missing data on age, sex, weight, height, blood pressure, serum bicarbonate, or serum creatinine were excluded. After the further exclusion of 1,910 screenings of participants younger than 20 years of age and with an eGFR of less than 60 mL/min per 1.73 m^2^, we analyzed the health records of the first screening visit of 41,886 adults. The study protocol was approved by the Institutional Review Board of Seoul National University Hospital.

Information on smoking status, alcohol ingestion, regular exercise, and previous history of diabetes, hypertension, and pharmacological treatment for diabetes and/or hypertension was obtained using a structured, self-report questionnaire and validated by direct interview with trained nurses. Trained physicians interviewed and examined all participants just before the health screening. The smoking status was classified according to three categories: current smokers, ex-smokers, and non-smokers. Participants who smoked at least one cigarette per day at the time of the health screening were categorized as current smokers. Participants who reported that they did not smoke at the time of the screening, but who used to smoke were categorized as ex-smokers. Regular drinkers were defined as participants who consumed alcoholic beverages at least once a week. Regular exercise was defined as exercise lasting longer than 30 min at least three times per week.

All the participants visited the hospital after an overnight fast. Body mass index (BMI) was calculated by dividing weight (kg) by height (m) squared. Blood pressure (BP) was measured using an automated BP-measuring device (Jawon, Busan, Korea) after resting in a sitting position for at least five minutes.

Blood samples and a urine specimen were taken after an overnight fast. Serum chemistry including bicarbonate and creatinine were measured using a Toshiba 200FR, a spectrophotometric chemistry auto analyzer (Toshiba Medical Systems Co., Tokyo, Japan). Serum creatinine was measured using the Jaffé method. To adjust the serum creatinine measurements to isotope dilution mass spectrophotometry, we reduced the serum creatinine levels by 5 % as previously recommended [14]. The participant’s eGFR was calculated with the Chronic Kidney Disease Epidemiology Collaboration equation (CKD-EPI) [15]. Serum bicarbonate was measured with enzymatic method using venous serum. The normal range of the bicarbonate of the venous serum in Seoul National University Hospital was 24–31 mEq/L. Albuminuria and urine pH were determined with a single spot urine dipstick analysis (YD Diagnostics, Yong-In, Korea), which was performed on morning urine samples after overnight fasting and was reported as negative, trace, 1+, 2+, 3+, or 4+. Albuminuria was defined as 1+ or higher. Urine pH was reported as 5.0, 5.5, 6.0, 6.5, 7.0, 7.5, 8.0, or 8.5. The participants who underwent health screening between Jan 2001 and May 2006 had lean mass evaluation using Inbody 2.0 (Biospace, Seoul, Korea) [16].

### Statistical analysis

Statistical analysis was conducted with R software 3.0.2 (http://www.R-project.org). The distribution of clinical parameters across the quintile groups of serum bicarbonate was compared with an ANOVA test for continuous variables and a chi-square test for discrete variables.

RHF was defined as previously suggested with some modification [17]. Briefly, the residuals were calculated from a multiple linear regression analysis, where logarithm-transformed eGFR was the dependent variable, while sex, height, weight, previous history of medication for diabetes and/or hypertension, and logarithm-transformed age were independent variables. An eGFR with a residual larger than the sex-specific 95th percentile was defined as RHF. The association between RHF and the quintile groups of serum bicarbonate was estimated with logistic regression analysis, adjusted for age, sex, smoking status, regular exercise, alcohol ingestion, history of therapy for diabetes and/or hypertension, BMI, systolic BP, fasting serum glucose, serum uric acid, serum calcium, serum albumin, serum triglyceride, serum high-density lipoprotein cholesterol, and albuminuria. The point-wise estimates and confidence intervals of odds ratio curves were computed with a multivariate logistic regression method adjusted for age, sex, BMI, systolic BP, alcohol intake, smoking status, regular exercise, previous medication for hypertension and/or diabetes, serum fasting glucose, serum uric acid, serum calcium, serum albumin, serum triglyceride, serum high-density lipoprotein cholesterol, and albuminuria. Penalized splines were used as the smoothing technique and the spline degrees of freedom were selected on the basis of the lowest Akaike Information Criterion. Two-sided p values less than 0.05 were considered statistically significant.

## Results

Table 1 describes the general characteristics of the participants, who had a mean age 49.5 years and of whom 51.0 % were male. The proportion of men, the mean age, the proportion of participants exercising regularly increased along with the increasing bicarbonate quintile level. The proportion of current smokers increased according to the decreasing serum bicarbonate quintile. The percentage of overweight participants with a BMI higher than 25 kg/m^2^ decreased from 35.7 % in the 1^st^ quintile to 29.8 % in the 5^th^ quintile (Table 1).

**Table 1:** General characteristics of the participants^a^

Serum bicarbonate quintile	1^st^	2^nd^	3^rd^	4^th^	5^th^	Total	*p* value^b^
(mEq/L)	(≤26)	(27–28)	(29)	(30–31)	(≥32)		
(*n*)	(9,745)	(11,016)	(5,999)	(9,351)	(5,775)	(41,886)	
Characteristics							
Men (%)	47.5	49.7	50.3	53.4	56.5	51.0	<.0001
Age at screening (years)	48.3 ± 0.2	49.3 ± 0.2	50.1 ± 0.3	50.9 ± 0.2	52.0 ± 0.3	49.9 ± 0.1	<.0001
The older (%)^c^	40.7	45.4	48.5	52.0	56.9	47.8	<.0001
Smoking (%)							<.0001
Nonsmoker	54.2	55.4	56.3	54.7	54.0	54.9	
Ex-smoker	18.2	20.2	20.9	23.2	25.3	21.2	
Current smoker	27.6	24.4	22.8	22.0	20.7	23.9	
Regular exercise (%)	36.4	38.7	39.1	39.9	43.3	39.1	<.0001
Regular alcohol intake (%)	48.3	50.3	49.8	51.6	49.6	49.9	.0044
History of anti-HT^d^ medication (%)	16.8	18.7	18.5	19.2	20.5	18.6	<.0001
History of DM^e^ medication (%)	6.3	6.7	6.6	7.4	9.0	7.1	<.0001
Systolic blood pressure (mmHg)	128.9 ± 0.4	129.1 ± 0.3	129.0 ± 0.5	129.4 ± 0.4	129.9 ± 0.5	129.2 ± 0.2	.0091
Diastolic blood pressure (mmHg)	78.5 ± 0.2	77.9 ± 0.2	77.5 ± 0.3	77.6 ± 0.2	77.9 ± 0.3	77.9 ± 0.1	<.0001
BMI^f^ (kg/m^2^)	24.0 ± 0.1	23.9 ± 0.1	23.9 ± 0.1	23.8 ± 0.0	23.6 ± 0.1	23.9 ± 0.0	<.0001
Overweight^g^ (%)	35.7	34.0	34.0	32.3	29.8	33.4	<.0001
Fasting serum glucose (mg/dL)	97.2 ± 0.5	96.4 ± 0.4	96.0 ± 0.5	96.4 ± 0.4	97.5 ± 0.6	96.7 ± 0.2	.0007
Serum albumin (mg/dL)	4.33 ± 0.01	4.37 ± 0.00	4.39 ± 0.01	4.41 ± 0.01	4.42 ± 0.01	4.38 ± 0.00	<.0001
Serum uric acid (mg/dL)	5.07 ± 0.03	5.11 ± 0.03	5.14 ± 0.03	5.17 ± 0.03	5.19 ± 0.03	5.13 ± 0.01	<.0001
Serum calcium (mg/dL)	9.29 ± 0.01	9.29 ± 0.01	9.31 ± 0.01	9.33 ± 0.01	9.37 ± 0.01	9.31 ± 0.00	<.0001
Serum total cholesterol (mg/dL)	196.4 ± 0.7	197.5 ± 0.7	199.8 ± 0.9	201.1 ± 0.7	202.3 ± 0.9	199.0 ± 0.3	<.0001
Serum triglyceride (mg/dL)	133.6 ± 1.9	128.9 ± 1.6	130.9 ± 2.3	131.4 ± 1.7	131.7 ± 2.2	131.2 ± 0.9	.0051
Serum HDL-cholesterol^h^(mg/dL)	65.3 ± 0.6	68.5 ± 0.7	69.4 ± 0.9	68.2 ± 0.7	67.4 ± 0.8	67.7 ± 0.3	<.0001
Albuminuria^i^ (%)	7.4	7.4	7.1	7.4	6.2	7.2	.0455
Acidic Urine pH^j^ (%)	46.3	36.0	30.7	24.7	17.3	32.5	<.0001
eGFR^k^ (mL/min/1.73m^2^)	84.0 ± 0.3	84.1 ± 0.2	83.4 ± 0.3	82.2 ± 0.3	80.4 ± 0.3	83.0 ± 0.1	<.0001
RHF^l^ (%)	4.6	4.5	4.8	3.2	2.9	4.1	<.0001

^a^Data represent mean ± standard deviation or proportion

^b^Based on ANOVA for continuous variables and chi-square test for discrete variables

^c^Older than median age at screening, 51 years for women and 50 years for men

^d^Hypertension

^e^Diabetes mellitus

^f^Body Mass Index

^g^BMI >25kg/m^2^

^h^Serum high-density lipoprotein cholesterol

^i^Spot urine dipstick test for albuminuria 1+ or higher

^j^Urine pH ≤5.5

^k^Estimated glomerular filtration rate calculated with the Chronic Kidney Disease Epidemiology Collaboration creatinine equation

^l^Renal hyperfiltration, see Methods for details

**Fig. 1:**
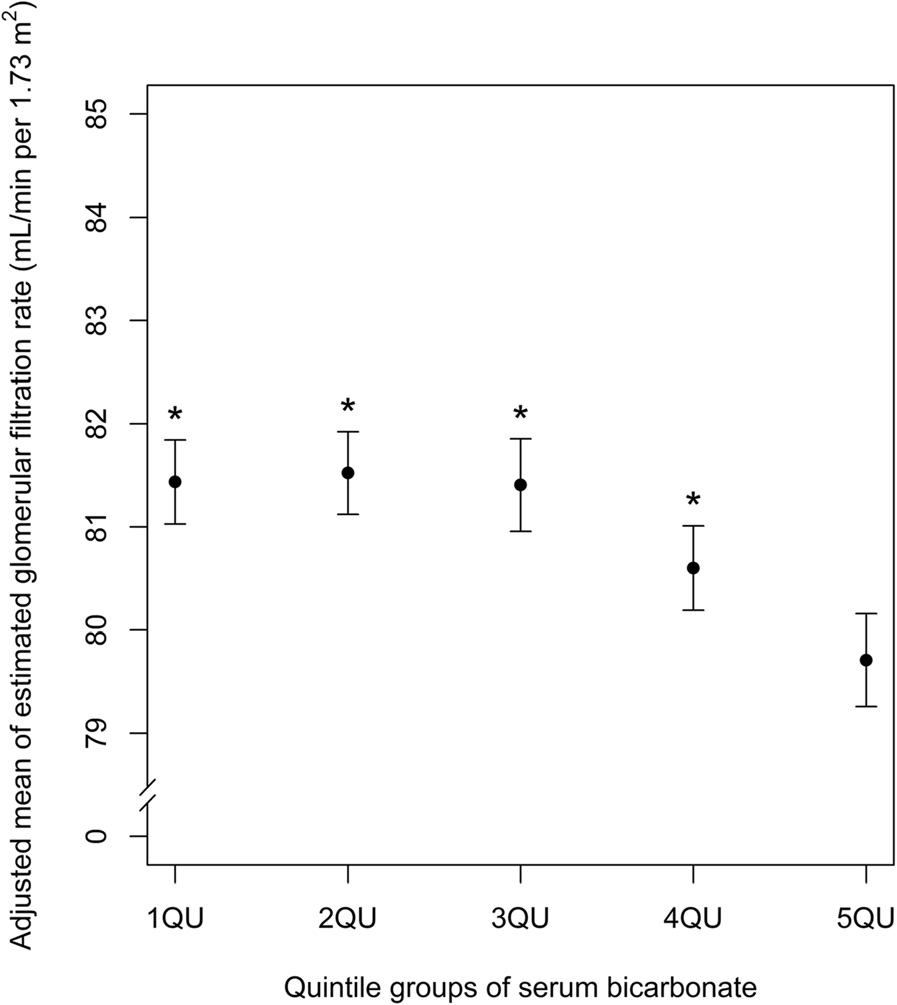
The adjusted mean of estimated glomerular filtration rate according to the quintile groups of serum bicarbonate level using a generalized linear model, after adjustment for possible confounding variables (see Methods for details). The error bars represent 95 % confidence intervals of the adjusted mean and asterisks indicate significant difference from the highest quintile group of serum bicarbonate level (*p* < 0.0001)

The adjusted mean eGFR of the highest quintile of serum bicarbonate was significantly lower than that of the other quintiles (adjusted mean eGFR 81.4 mL/min per 1.73 m^2^, 95 % confidence interval 81.0–81.8 mL/min per 1.73 m^2^ in the first quintile; 81.5, 81.1–81.9 in the second quintile, 81.4, 81.0–81.9 in the third quintile, 80.6, 80.2–81.0 in the fourth quintile, 79.7, 79.3–80.2 in the highest quintile) after adjustment for age, sex, smoking status, regular exercise, alcohol ingestion, history of pharmacological therapy for hypertension and/or diabetes, BMI, systolic BP, fasting serum glucose, serum uric acid, serum calcium, serum albumin, serum triglyceride, serum high-density lipoprotein cholesterol, and albuminuria (*p* < 0.0001 the highest quintile vs the other quintiles by generalized linear model; Fig. 1).

**Fig. 2:**
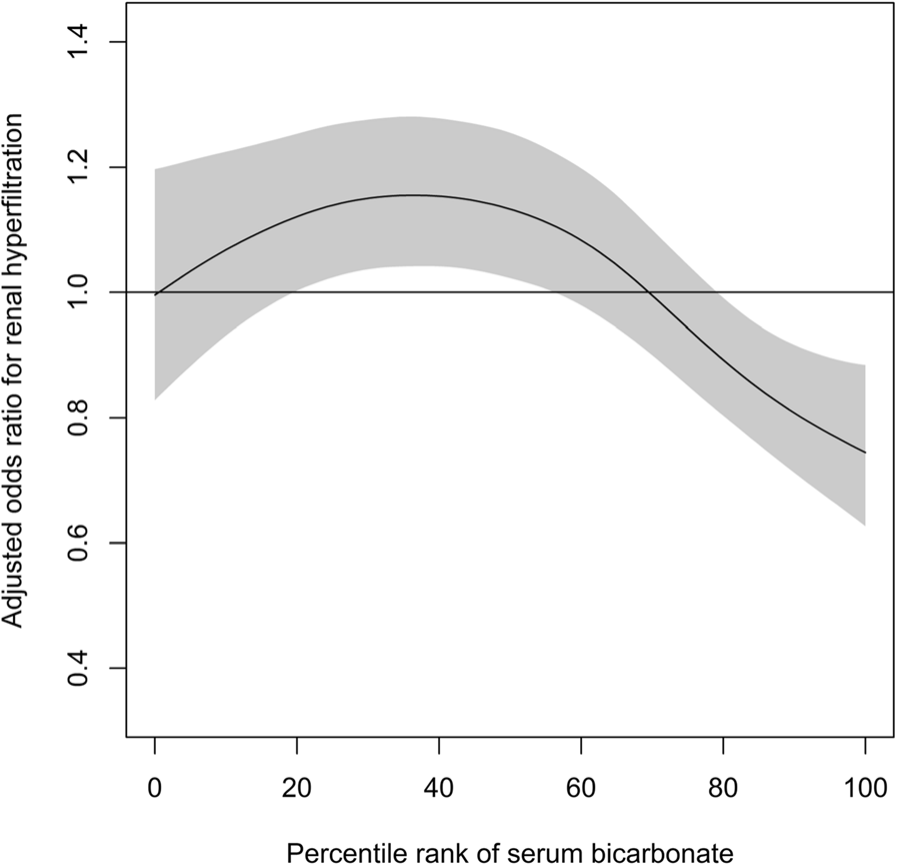
Association between the percentile rank of serum bicarbonate level and the odds of renal hyperfiltration after adjustment for possible confounding variables (see Methods for details). A multivariate logistic regression model was used to compute point-wise estimates and confidence intervals of odds ratio curves. The solid line represents the adjusted odds ratio and the shaded area the 95 % confidence interval

The association between the distribution of serum bicarbonate and the odds of RHF, defined as an eGFR with an adjusted residual higher than the 95^th^ percentile, was analyzed with multivariate logistic regression analysis, adjusted for age, sex, smoking status, regular exercise, alcohol ingestion, history of pharmacological therapy for hypertension and/or diabetes, BMI, systolic BP, fasting serum glucose, serum uric acid, serum calcium, serum albumin, serum triglyceride, serum high-density lipoprotein cholesterol, and albuminuria. The lower percentile rank of serum bicarbonate was associated with higher odds of RHF (Fig. 2). With multivariate logistic regression analysis, the odds of RHF in the lower quintile groups of serum bicarbonate were higher than that in the highest quintile group (odds ratio 1.39, 95 % confidence interval 1.11–1.75 in the lowest quintile, 1.41, 1.13–1.76 in the second quintile, 1.55, 1.22–1.98 in the third quintile, compared to the highest quintile; Table 2). After exclusion of the participants taking anti-hypertensive medications, the association between RHF and serum bicarbonate was similar (odds ratio 1.40, 95 % confidence interval 1.11–1.77 in the lowest quintile, 1.38, 1.10–1.74 in the second quintile, 1.55, 1.21–1.99 in the third quintile, compared to the highest quintile; data not shown). After exclusion of the participants with fasting serum glucose above 125 mg/dL, the association between RHF and serum bicarbonate was significant (odds ratio 1.26, 95 % confidence interval 1.00–1.58 in the lowest quintile, 1.32, 1.06–1.75 in the second quintile, 1.55, 1.22–1.97 in the third quintile, compared to the highest quintile; data not shown).

**Table 2:** Association between serum bicarbonate level and renal hyperfiltration

		Quintile groups of serum bicarbonate	Renal hyperfiltration^a^ (%)	Odds ratio (95 % confidence interval) for renal hyperfiltration^b^	*p* for interaction
Total		1^st^	300/6550 (4.6)	1.39 (1.11 to 1.75)	
		2^nd^	322/7106 (4.5)	1.41 (1.13 to 1.76)	
		3^rd^	188/3885 (4.8)	1.55 (1.22 to 1.98)	
		4^th^	193/6110 (3.2)	1.02 (0.81 to 1.30)	
		5^th^	111/3801 (2.9)	Reference	
Sex	Men	1^st^	128/3294 (3.9)	1.40 (1.01 to 1.93)	0.9999
		2^nd^	145/3715 (3.9)	1.39 (1.02 to 1.97)	
		3^rd^	87/2067 (4.2)	1.51 (1.08 to 2.26)	
		4^th^	95/3411 (2.8)	1.00 (0.72 to 1.46)	
		5^th^	59/2235 (2.6)	Reference	
	Women	1^st^	172/3256 (5.3)	1.39 (1.01 to 1.93)	
		2^nd^	177/3391 (5.2)	1.43 (1.04 to 1.97)	
		3^rd^	101/1818 (5.6)	1.60 (1.13 to 2.26)	
		4^th^	98/2699 (3.6)	1.03 (0.73 to 1.46)	
		5^th^	52/1566 (3.3)	Reference	
Age	Young	1^st^	194/3839 (5.1)	1.66 (1.18 to 2.32)	0.6341
		2^nd^	192/3878 (5.0)	1.68 (1.21 to 2.35)	
		3^rd^	105/2040 (5.1)	1.81 (1.27 to 2.60)	
		4^th^	98/2986 (3.3)	1.18 (0.82 to 1.69)	
		5^th^	45/1704 (2.6)	Reference	
	Older^c^	1^st^	106/2711 (3.9)	1.25 (0.91 to 1.72)	
		2^nd^	130/3228 (4.0)	1.25 (0.92 to 1.70)	
		3^rd^	83/1845 (4.5)	1.39 (1.00 to 1.94)	
		4^th^	95/3124 (3.0)	0.93 (0.68 to 1.28)	
		5^th^	66/2097 (3.1)	Reference	
BMI^d^	≤25 kg/m^2^	1^st^	168/4132 (4.1)	1.18 (0.89 to 1.56)	0.0907
		2^nd^	199/4581 (4.3)	1.31 (1.00 to 1.71)	
		3^rd^	113/2528 (4.5)	1.40 (1.04 to 1.88)	
		4^th^	134/4066 (3.3)	1.04 (0.78 to 1.39)	
		5^th^	79/2643 (3.0)	Reference	
	>25 kg/m^2^	1^st^	132/2418 (5.5)	1.98 (1.32 to 2.95)	
		2^nd^	123/2525 (4.9)	1.72 (1.15 to 2.57)	
		3^rd^	75/1357 (5.5)	1.98 (1.30 to 3.03)	
		4^th^	59/2044 (2.9)	1.03 (0.66 to 1.60)	
		5^th^	32/1158 (2.8)	Reference	

^a^See Methods for details

^b^By multivariate logistic regression analysis, adjusted for systolic blood pressure, alcohol intake, smoking status, regular exercise, medication for hypertension and/or diabetes, serum fasting glucose, serum uric acid, serum calcium, serum albumin, serum triglyceride, serum high density lipoprotein-cholesterol, and albuminuria, where age, sex, or body mass index were added as adjusted variables except for the variable of interest

^c^Older than the sex-specific median age, 51 years for women and 50 years for men

^d^Body Mass Index

With subgroup analysis, the association between serum bicarbonate level and the odds of RHF was significant only in participants with a BMI higher than 25 kg/m^2^ compared to those with a BMI of 25 kg/m^2^ or lower, although the p value for interaction did not reach the statistical significance (Table 2). The odds ratio of the lowest quintile was 1.98 (95 % confidence interval 1.32–2.95) compared to the highest quintile in participants with a BMI higher than 25 kg/m^2^, and was 1.18 (95% confidence interval 0.89–1.56) in those with a BMI of 25 kg/m^2^ or lower (Table 2). Urine pH and serum anion gap were not associated with the odds of RHF (data not shown).

## Discussion

This study, using a very large sample size, observed that a lower serum bicarbonate level was associated with increased odds of RHF, defined as an eGFR with adjusted residuals above the sex-specific 95^th^ percentile. This observation was more prominent in subjects with a BMI above 25 kg/m^2^ than in the counterpart subgroup with a BMI below 25 kg/m^2^, although the results of the subgroup analysis did not reach the statistical significance.

RHF has been associated with many clinical conditions and lifestyle factors considered to increase the risk of CKD or cardiovascular diseases [4–11]. RHF has long been hypothesized as one of the main mechanisms of renal disease progression, irrespective of the underlying causes of CKD and the measures alleviating RHF, such as restriction of dietary protein and salt intake and anti-hypertensive therapy, especially the renin-angiotensin-aldosterone system blockers, have been recommended to patients with CKD [18]. Recently, the clinical implication of RHF has been reported to be beyond the scope of kidney diseases and the possibility of RHF as a predictor of all-cause and cardiovascular mortality risk in the general population has been suggested [12]. But the clarification is still needed on the mechanism(s) underlying this association.

The modern Western-type diet is characterized by a deficiency of fruit and vegetables and excessive consumption of animal products, and the metabolism of sulfur-containing amino acids such as methionine, homocysteine, and cysteine in animal proteins and cereal grains generates sulfate, a non-metabolizable anion constituting a major determinant of the daily acid load [13]. All the subjects in this study had an eGFR higher than 60 mL/min per 1.73 m^2^ where the excretion of daily acid load should not be abnormal, and the lower serum bicarbonate level observed in a number of these subjects could be assumed to be resulting from dietary causes [19].

Subclinical metabolic acidosis can cause various renal adaptive responses, including RHF. Metabolic acidosis due to an acidogenic diet has been hypothesized to cause insulin resistance, metabolic syndrome, and hence cardiovascular risk [13]. Although subclinical chronic metabolic acidosis may be a possible mechanism explaining the association between RHF and increased mortality, this possibility has not been tested as yet. Recently, a lower serum bicarbonate level has also been reported as an all-cause and cardiovascular mortality risk factor in the general population with preserved renal function [20]. The association between RHF and the lower serum bicarbonate level observed in this study may suggest the possibility of metabolic acidosis as a mechanism linking RHF and increased mortality risk. The observation that the association between RHF and the lower serum bicarbonate level was more prominent in the subjects with BMI higher than 25 kg/m^2^ may be indirect evidence of the linkage between metabolic acidosis, metabolic syndrome, and cardiovascular risk. Recently, the association between low serum bicarbonate and a higher risk of incident CKD in the general population with preserved renal function has been reported. Complement activation and tubulointerstitial injury due to a compensatory increase in ammoniagenesis in the remaining nephrons, a marker of tubular dysfunction, and a marker of dietary habits such as lower intake of fruit and vegetables have been proposed as possible explanations [2, 3]. As RHF has been associated with a rapid decline in eGFR in a population with preserved renal function [12], the association between low serum bicarbonate and RHF may be another explanation for the association between lower serum bicarbonate and incident CKD.

Clarification is still needed regarding the mechanism(s) underlying the association between the low serum bicarbonate level and RHF. It is well known that high protein intake can increase the glomerular filtration rate in human beings and that substitution of soy protein for animal protein results in less hyperfiltraton [21]. In a cross-sectional study of 2,938 CKD patients, the percentage of total protein intake from plant sources was associated with a higher serum bicarbonate level [22]. An explanation for the differential effect of the sources of protein in RHF is not available. It has been suggested that chronic acidosis, chronic potassium deficiency, and chronic hyperfiltration share a common signaling pathway to the demand for increased hydrogen transport [23]. Therefore, the hypothesis that a higher dietary acid load mainly from animal proteins and cereals decreases the serum bicarbonate level and thereby causes RHF needs to be tested through randomized controlled studies.

The association between RHF and serum bicarbonate was not linear. This can be explained by the activation of counter-balancing mechanism(s) such as tubuloglomerular feedback. An S-shaped relationship between loop of Henle perfusion rate and single nephron GFR in rat has been reported [24]. This hypothesis needs to be confirmed.

Contrary to serum bicarbonate levels, either of serum anion gap and urine pH was not associated with RHF in this study. The explanation for this lack of association is not clear. Higher dietary sodium intake has been reported to be associated with hyperchloremic metabolic acidosis [25] and RHF [26] in general population. In Korea, the mean dietary sodium intake per person was 4.6–4.7 g/day, higher than that in Western countries [27]. The lack of association between serum anion gap and RHF can be explained by the confounding effect of higher dietary sodium intake. This hypothesis needs to be confirmed by future studies. The measurement of urine pH in this study was done by dipstick test on voided urine not on 24-h urine collection. The first void urine pH by paper strip has been reported not being a reliable indicator of daily net endogenous acid production [28]. The association between urine pH and RHF may need to be analyzed with urine pH measured using 24-h urine collection.

It can be argued that volume depletion caused by diuretic medications can confound the association between RHF and serum bicarbonate. After exclusion of the participants taking anti-hypertensive medications, the association was similar and the possibility of confounding effect of diuretic medication can be reasonably excluded. Although hyperglycemia may influence the prevalence of RHF in the quintile groups of serum bicarbonate, the association between RHF and serum bicarbonate was independent of fasting serum glucose. After exclusion of the participants with fasting serum glucose above 125 mg/dL, the association was still significant. Therefore, the possibility of confounding effect of hyperglycemia in this association seems to be quite low. The subset of the participants who underwent health screening between Jan 2001 and May 2006 measured lean mass with bioimpedance analysis [16]. With inclusion of lean mass quartile in the multivariate logistic regression model, the association between RHF and serum bicarbonate was independent of the lean mass quartiles. Therefore, the confounding effect of skeletal muscle protein metabolism caused by chronic metabolic acidosis on the association between RHF and serum bicarbonate could be excluded.

With subgroup analysis, the association between the serum bicarbonate quintile groups and RHF was significant only in subjects with a BMI higher than 25 kg/m^2^, although the p value for interaction was not significant. A possible differential effect according to BMI can suggest the pathophysiological roles of obesity in the association between serum bicarbonate and RHF [20]. Although the association between metabolic acid load and type 2 diabetes or hypertension has been observed only in studies of women [29–33], the current study did not observe a gender effect in the association between lower serum bicarbonate and RHF. Future studies on the possible gender effect in the association between metabolic acid load and RHF are necessary.

There are some weaknesses in this study. First, the study design was cross-sectional and the causal relationship between lower serum bicarbonate level and RHF could not be conclusively determined. Prospective studies may provide definite evidence. Second, the results of this study emerged from observations in a single hospital and with a population consisting of a single ethnic origin. Therefore, any extrapolation of the results to other ethnic groups should be treated with caution. Third, the measurement of serum bicarbonate was performed only once. The subjects visited the hospital for a routine health screening, not on account of their illnesses, and the measurements could be assumed to be representative of the stable condition of the participants on a regular diet. Forth, the data on the hydration status of the participants were not available. Although the participants of this study were relatively healthy and supposed to be in a stable condition, the confounding effect of the volume status on the association between RHF and serum bicarbonate observed in this study could not be excluded clearly. The duration of fasting was usually shorter than 12 h and supposed to be similar across the quintile groups of serum bicarbonates. Therefore, the degree of volume depletion and its impact on the association between RHF and serum bicarbonate might be minimal. Fifth, GFR was estimated, not measured. Lastly, the data on the duration of diabetes of the participants was not available. Although the association was independent of fasting serum glucose, the effect of the duration of diabetes on the lower prevalence of RHF in the quintile groups with higher serum bicarbonate levels could not be excluded clearly. Despite these limitations, to the best of our knowledge, this study observed for the first time the association between lower serum bicarbonate level and RHF in a relatively large population with preserved renal function sufficient to excrete a daily acid load.

## Conclusions

In conclusion, in a very large sample size lower serum bicarbonate was associated with higher odds of RHF, and with subgroup analysis, the possible differential effects of obesity have been suggested. These observations can provide clues for the study of the pathophysiology of the linkage between dietary factors, CKD and cardiometabolic risk, and for the development of dietary guidelines to prevent CKD or cardiovascular diseases. It is necessary to confirm the association between lower serum bicarbonate level and RHF and its causality through prospective studies.

## Abbreviations

BMI: body mass index
BP: blood pressure
CI: confidence interval
CKD: chronic kidney disease
CKD-EPI: Chronic Kidney Disease Epidemiology Collaboration equation
DM: diabetes mellitus
eGFR: estimated glomerular filtration rate
HDL-cholesterol: high-density lipoprotein cholesterol
HT: hypertension
OR: odds ratio
RHF: renal hyperfiltration
